# An artificial neural network model based on standing lateral radiographs for predicting sitting pelvic tilt in healthy adults

**DOI:** 10.3389/fsurg.2022.977505

**Published:** 2022-09-14

**Authors:** Minwei Zhao, Yuanbo He, Shuai Li, Huizhu Chen, Weishi Li, Hua Tian

**Affiliations:** ^1^ Department of Orthopedic Surgery, Peking University Third Hospital, Beijing, China; ^2^ Engineering Research Center of Bone and Joint Precision Medicine, Ministry of Education, Beijing, China; ^3^ State Key Laboratory of Virtual Reality Technology and Systems, Beihang University, Beijing, China

**Keywords:** spinopelvic motion, sagittal plane, standing and sitting, total hip arthroplasty, Pearson correlation analysis

## Abstract

**Background:**

Spinopelvic motion, the cornerstone of the sagittal balance of the human body, is pivotal in patient-specific total hip arthroplasty.

**Purpose:**

This study aims to develop a novel model using back propagation neural network (BPNN) to predict pelvic changes when one sits down, based on standing lateral spinopelvic radiographs.

**Methods:**

Young healthy volunteers were included in the study, 18 spinopelvic parameters were taken, such as pelvic incidence (PI) and so on. First, standing parameters correlated with sitting pelvic tilt (PT) and sacral slope (SS) were identified *via* Pearson correlation. Then, with these parameters as inputs and sitting PT and SS as outputs, the BPNN prediction network was established. Finally, the prediction results were evaluated by relative error (RE), prediction accuracy (PA), and normalized root mean squared error (NRMSE).

**Results:**

The study included 145 volunteers of 23.1 ± 2.3 years old (M:F = 51:94). Pearson analysis revealed sitting PT was correlated with six standing measurements and sitting SS with five. The best BPNN model achieved 78.48% and 77.54% accuracy in predicting PT and SS, respectively; As for PI, a constant for pelvic morphology, it was 95.99%.

**Discussion:**

In this study, the BPNN model yielded desirable accuracy in predicting sitting spinopelvic parameters, which provides new insights and tools for characterizing spinopelvic changes throughout the motion cycle.

## Introduction

**Figure 1:**
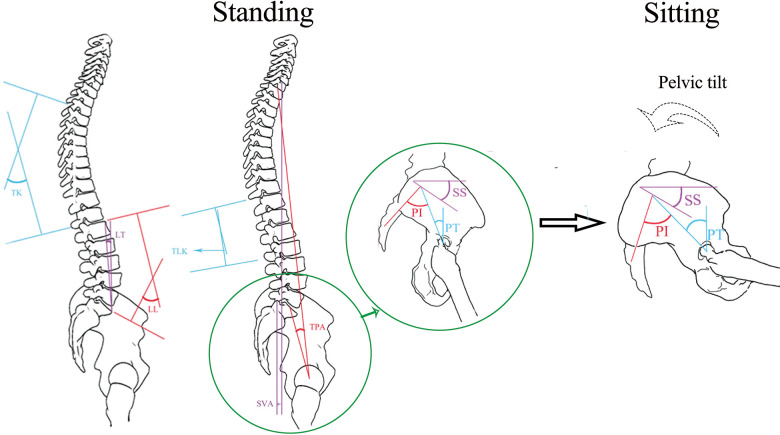
As the body posture changes from standing to sitting, the spinopelvic coordination maintains the sagittal balance of the body posture. See the “Parameter measurement” section for the description of spinopelvic parameters (PI, PT, SS, TK, LL, LT, TLK, TPA, and T1PA).

As shown in Figure 1, the spinopelvic coordination maintains the sagittal balance of body posture. It enables straightened lumbar spine and posterior pelvic tilt in the sitting position to accommodate flexion and internal rotation of the femur and prevent anterior impingement and posterior dislocation in normal physiology. In the standing posture, in contrast, it allows increased lumbar lordosis and anterior pelvic tilt to increase acetabular coverage, thus preventing posterior impingement and anterior dislocation (1).

The traditional safe zone for cup position in total hip arthroplasty (THA), definitive treatment for advanced hip arthritis, has been based on a vertically oriented anterior pelvic plane (2). Therefore, it does not account for the spinopelvic balance of each individual or the change of pelvic tilt in various body postures (3). As a result, some patients may engage in more aggressive hip motions to maintain sagittal balance when they change position from sitting to standing. This abnormality may lead to secondary dislocation and impingement, and the resulting edge loading compromises prosthesis survivorship. This condition becomes more severe with concomitant lumbar spine diseases (4). Thus, surgeons should consider the sagittal spinopelvic balance when planning for THA (5). Unfortunately, although it is an increasingly accepted concept, studies on solutions are scanty (6).

Artificial neural network (ANN), investigating correlation among subjects, has been attempted in prognostication and drug discovery with demonstrated accuracy and robustness (7). Composed of musculoskeletal and ligament structures and controlled by neuromuscular interaction, the spinopelvic system engages in coordinated sagittal motion with the essential correlation of spinopelvic features between standing and sitting positions. Using the back propagation neural network (BPNN) and standing lateral spinopelvic radiographs of healthy volunteers, this study aims to predict how the pelvic tilts when the human body changes position from standing to sitting. The results of this study will pave the way for characterizing the dynamics of the spinopelvic system at various positions along the motion cycle.

## Materials and methods

### Study type

This prospective study has been approved by the Ethics Committee of our institution (project number IRB00006761–2012066). All volunteers provided written informed consent.

### Eligibility criteria

The inclusion criterion was participants should be between 18 and 30 years old. The exclusion criteria were as follows: (1) chronic lower back and leg pain, spine deformity, and history of disease or surgery of the spine, pelvis, hip joint, and lower limbs; and (2) spondylolisthesis, scoliosis with a Cobb Angle >10°, and kyphosis on spinopelvic frontal and lateral radiographs.

### Radiographs

Radiographs in standard standing and sitting positions of the whole spine and pelvis, including bilateral hip joints, were obtained from all research subjects. Participants were asked to stand as straight as possible in the standard standing position without leaning forward or backward. In the standard sitting position, they were asked to remain seated as straight as possible, without leaning forward or backward, and with both knees and hips flexed at 90°. For improved quality of the x-ray film, the elbow joints were flexed fully, and the fists rested on the ipsilateral clavicle. After continuous exposure, the image was automatically spliced.

### Parameter measurement

Pelvic and spinal parameters, as shown in Figure 1, were measured in Picture Archiving and Communication Systems (Centricity RIS/PACS, GE Healthcare: https://www.gehealthcare.com/). All parameters were measured independently by two senior radiologists. They produced two readings from every image, then compared the results within (intraobserver) and between themselves (interobserver), and took the average value as the final result. The following parameters were measured in both standing and sitting position radiographs: (1) pelvic incidence (PI): the angle between the line perpendicular to the sacral plate at its midpoint and the line connecting the same point to the center of the bicoxofemoral axis; (2) pelvic tilt (PT): the angle between the line connecting the midpoint of the sacral plate to the center of the bifemoral heads, and the plumb line; (3) sacral slope (SS): the angle between the sacral plate and the horizontal; (4) thoracic kyphosis (TK): the angle between the upper endplate of T4 and the lower endplate of T12; (5) lumbar lordosis (LL): the angle between the upper endplate of L1 and the upper endplate of S1; (6) lordosis tilt (LT): the angle between the line connecting the anterosuperior margin of L1 to the anterosuperior margin of S1, and the plumb line; (7) thoracolumbar kyphosis (TLK): the angle between the upper endplate of T11 and the lower endplate of L2; (8) T1 pelvic angle (TPA): the angle between the line connecting the midpoint of the upper endplate of T1 and the center of the bifemoral heads, and the line connecting the midpoint of the upper endplate of S1 and the center of the bifemoral heads; (9) sagittal vertical axis (SVA): the distance from the posterosuperior edge of the sacrum to the C7 plumb line.

### Statistical analysis

Statistical analyses were performed using SPSS software (version 18.0). Measurement data were expressed as mean ± SD (min-max), and Pearson correlation coefficient (8) was used for the correlation analysis. Values of *p* < 0.05 were considered to indicate a statistically significant difference.

### Back propagation neural network

**Figure 2:**
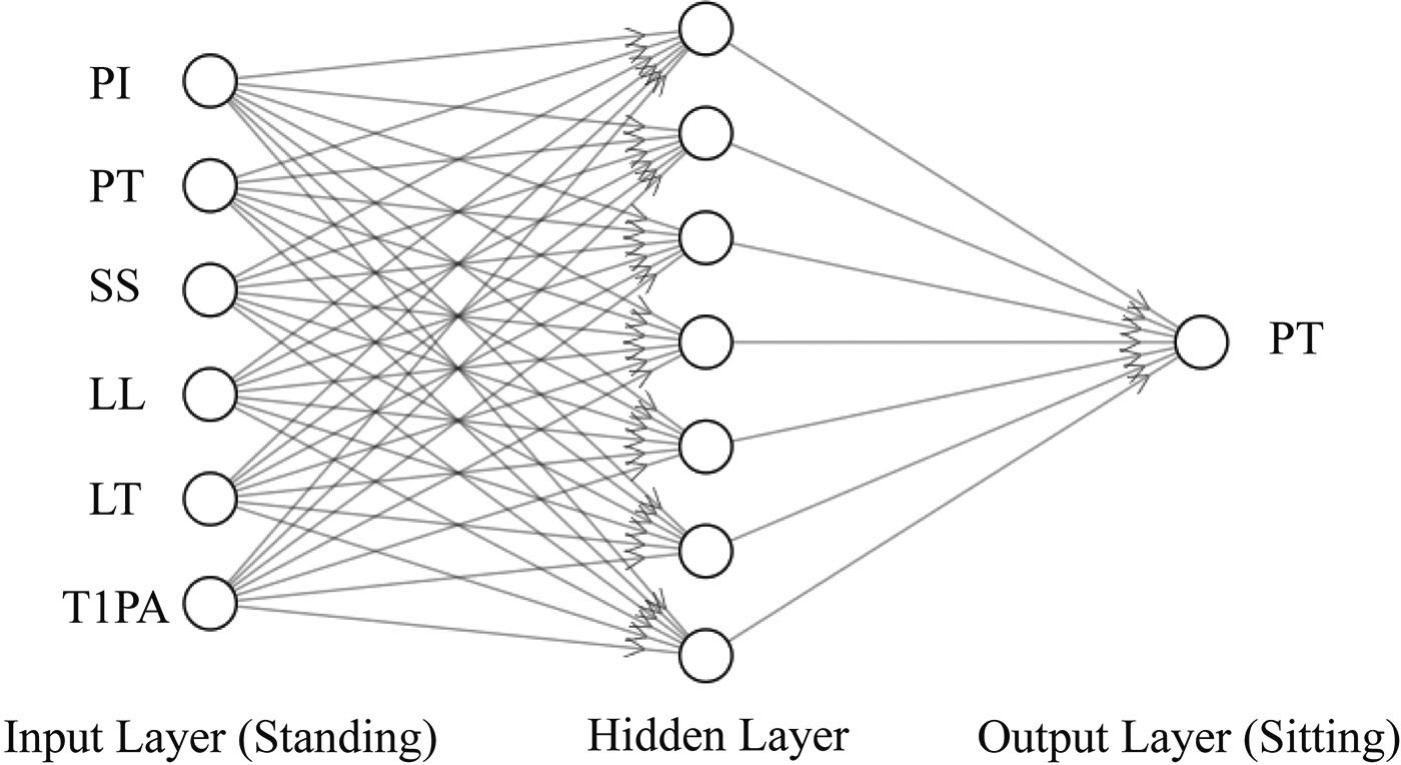
The BPNN framework for predicting the sitting PT with the standing parameters related to the sitting PT as input.

Input and output. Inputs were the correlated parameters identified in Pearson correlation analysis, as shown in Table 1. The sitting PT (PT in sitting position) was correlated to PI, PT, SS, LL, LT, and T1PA in standing position (as shown in the input layer of Figure 2). The sitting SS (SS in sitting position) was correlated to PI, SS, LL, LT, and SVA in standing position. Likewise, the sitting PI (PI in sitting position) was correlated to PI, PT, SS, LL, LT, TLK, and T1PA in standing position. Outputs were sitting PT, SS, and PI.

**Table 1:** Correlation between sitting PI, PT, SS, and various sitting parameters (*r*, *p*).

	Standing	Standing	Standing	Standing	Standing	Standing	Standing	Standing	Standing
	PI	PT	SS	LL	LT	TLK	TK	T1PA	SVA
Sitting PT	0.546,	0.472,	0.272,	0.216,	0.220,	−0.100,	−0.103,	0.420,	−0.023,
	*<*0.001	*<*0.001	0.001	0.009	0.008	0.234	0.218	*<*0.001	0.782
Sitting SS	0.371,	0.104,	0.383,	0.312,	0.264,	−0.074,	0.097,	0.160,	0.178,
	*<*0.001	0.212	*<*0.001	0.001	0.001	0.374	0.224	0.055	0.032
Sitting PI	0.955,	0.613,	0.670,	0.539,	0.504,	−0.190,	−0.018,	0.609,	0.147,
	*<*0.001	*<*0.001	*<*0.001	*<*0.001	0.001	0.022	0.834	*<*0.001	0.078

*Model theory*. The BPNN (9) was used to construct the nonlinear regression between input and output. As shown in Figure 2, taking predicting sitting PT as an example, the BPNN used standing PI, PT, SS, LL, LT, and T1PA as the input layer. To avoid overfitting due to the lack of training data (10), a single hidden layer with seven units was selected. The sitting PT was the output layer.

*Volunteer grouping*. A total of 145 volunteers with standing–sitting pelvic and spinal parameters were randomly divided into training, validation, and test sets according to *a priori* of 8:1:1. The training set was used to train the model and determine the model parameters. The validation set was used to adjust the model's super parameters and to preliminarily evaluate the model's ability. The test set was used to evaluate the generalizability of the final model.

*Training of the BPNN*. The training sample data were normalized and then inputted into the network. The activation functions of the hidden and output layers were set as tansig (hyperbolic tangent S type) and purelin (linear) functions, respectively. The training function of the network was trainlm, the performance function of the network was MSE (11), and the number of neurons in the hidden layer was initially set to 7. The number of network iterations was 5,000, with an expected error of 0.0000001 and a learning rate of 0.01. After setting the parameters, the training network was started, and the experiment platform used was Matlab (12) (2017a) + Windows 10.

*Verifying of the BPNN*. Training 10 times in the same way, the model with the best performance on the validation set was taken as the functional BPNN. After the functional BPNN was obtained, we verified the BPNN on the test set. The evaluation indicators were as follows: Relative Error (RE)=|predicted value−actual value|/actual value, Prediction Accuracy (PA) = 1-RE, and the Normalized Root Mean Squared Error (NRMSE) (13).

## Results

*General information*. A total of 145 volunteers (51 men, 94 women) of 23.1 ± 2.3(19–29) years old on average were recruited. Spinopelvic parameters in standing and sitting positions are described in Table 2.

**Table 2:** Spinopelvic parameters in standing and sitting positions [mean ± SD (min-max)].

Parameter	Standing	Sitting
PI (°)	46.6 ± 9.1 (25.6, 69.7)	48.0 ± 9.1 (25.6, 69.7)
PT (°)	11.8 ± 6.5 (−8.3, 27.6)	28.4 ± 10.0 (1.3, 53.0)
SS (°)	34.9 ± 7.1 (13.5, 52.3)	19.7 ± 8.7 (0.9, 42.0)
LL (°)	50.4 ± 10.0 (23.5, 72.9)	25.3 ± 11.8 (1.0, 54.7)
LT (°)	−5.0 ± 5.0 (−17.0, 7.7)	−1.8 ± 5.8 (−15.2, 11.9)
TLK (°)	6.3 ± 5.4 (0.1, 27.3)	6.6 ± 4.8 (0.1, 20.1)
TK (°)	26.1 ± 10.2 (2.4, 72.0)	20.0 ± 8.9 (0.7, 49.6)
T1PA (°)	5.6 ± 6.0 (−16.3, 18.7)	23.7 ± 9.3 (3, 49)
SVA (mm)	−20.1 ± 22.4 (−69.7, 74.2)	26.9 ± 28.6 (−45, 103)

*Correlation analysis*. As shown in Table 1, sitting PT (PT in sitting position) was correlated to PI, PT, SS, LL, LT, and T1PA in standing position. Likewise, sitting SS (SS in sitting position) was correlated to PI, SS, LL, LT, and SVA in standing position. Moreover, sitting PI (PI in sitting position) was correlated to PI, PT, SS, LL, LT, TLK, and T1PA in standing position.

**Figure 3:**
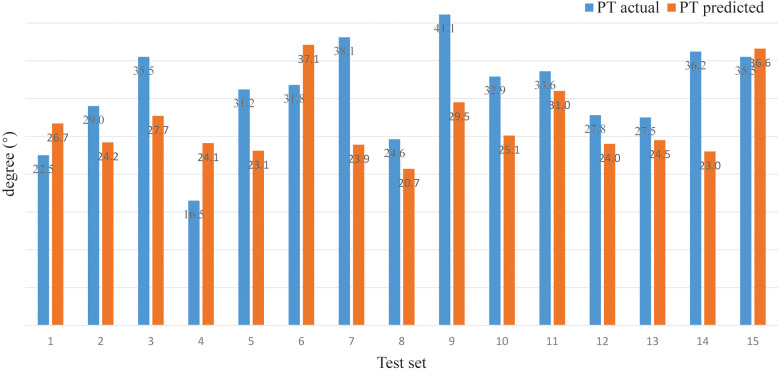
Comparison between the actual and the predicted values of sitting PT.

**Figure 4:**
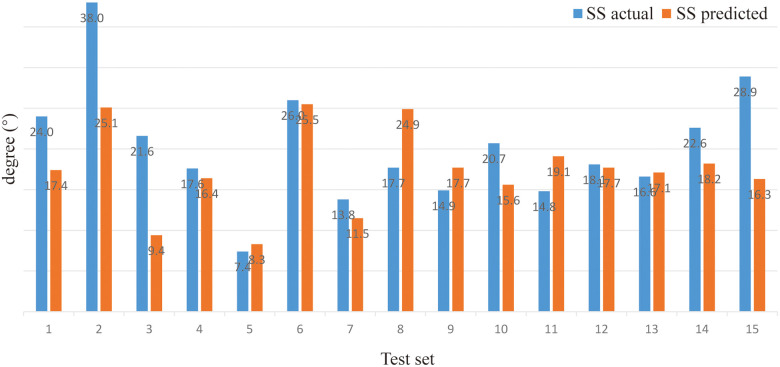
Comparison between the actual and the predicted values of sitting SS.

**Figure 5:**
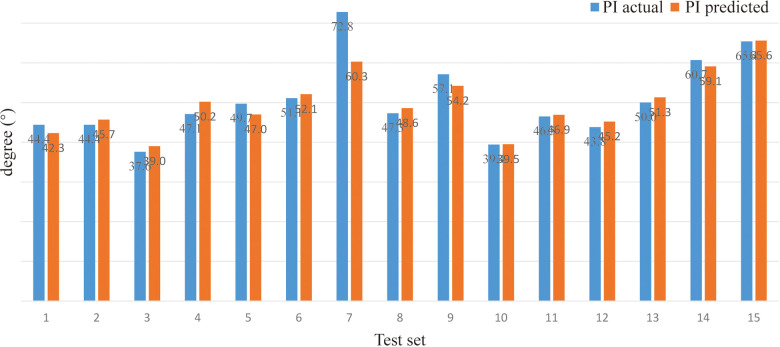
Comparison between the actual and the predicted values of sitting PI.

*Model prediction results* *(verifying of BPNN)*. For sitting PT, the PA of the BPNN model was 78.48% (RE = 21.52%, NRMSE = 13.95%) (Figure 3; Table 3). For sitting SS, the PA of the BPNN model was 71.17% (RE = 28.83%, NRMSE = 11.76%) (Figure 4; Table 3). For sitting PI, the PA of the BPNN model was 95.99% (RE = 4.01%, NRMSE = 4.09%) (Figure 5; Table 3). Compared with some simpler artificial models such as multi-linear regression (14), elastic net regression (15), and support vector regression (SVR) (16), the BPNN is better at dealing with complex nonlinear relationships in prediction. As outlined in Table 4, the BPNN achieves the best results by a clear margin. It indicates that the BPNN based on standing lateral radiographs for predicting sitting pelvic tilt in healthy adults is feasible and superior.

**Table 3:** Predicting sitting PT, SS, and PI based on the BPNN from standing parameters.

Test set	1	2	3	4	5	6	7	8	9	10	11	12	13	14	15	PA (%)	RE (%)	NRMSE (%)
PT (°)	Actual	22.5	29.0	35.5	16.5	31.2	31.8	38.1	24.6	41.1	32.9	33.6	27.8	27.5	36.2	35.5	**78** **.** **48**	**21** **.** **52**	**13** **.** **95**
	Predicted	26.7	24.2	27.7	24.1	23.1	37.1	23.9	20.7	29.5	25.1	31.0	24.0	24.5	23.0	36.6			
SS (°)	Actual	24.0	38.0	21.6	17.6	7.4	26.0	13.8	17.7	14.9	20.7	14.8	18.1	16.6	22.6	28.9	**71** **.** **17**	**28** **.** **83**	**11** **.** **76**
	Predicted	17.4	25.1	9.4	16.4	8.3	25.5	11.5	24.9	17.7	15.6	19.1	17.7	17.1	18.2	16.3			
PI (°)	Actual	44.4	44.4	37.6	47.1	49.7	51.1	72.8	47.3	57.1	39.4	46.5	43.8	50.0	60.7	65.4	**95** **.** **99**	**4** **.** **01**	**4** **.** **09**
	Predicted	42.3	45.7	39.0	50.2	47.0	52.1	60.3	48.6	54.2	39.5	46.9	45.2	51.3	59.1	65.6			

**Table 4:** The performance of different methods for predicting sitting PT, SS, and PI. The larger *PA* and smaller *RE* and *NRMSE*, the better the performance. Bold means the best.

	PT			SS			PI		
Methods	PA (%)	RE (%)	NRMSE (%)	PA (%)	RE (%)	NRMSE (%)	PA (%)	RE (%)	NRMSE (%)
Multilinear (1)	61.11	38.89	27.46	34.69	65.31	37.36	95.31	4.69	6.24
Elastic net (2)	59.10	40.90	27.92	36.29	63.71	36.96	94.44	5.56	7.06
SVR [3]	59.08	40.92	27.77	33.77	66.23	37.55	95.12	4.88	6.17
Ours (BPNN)	**78** **.** **48**	**21** **.** **52**	**13** **.** **95**	**71** **.** **17**	**28** **.** **83**	**11** **.** **76**	**95** **.** **99**	**4.**01	**4** **.** **09**

## Discussion

The spine and pelvis are characterized by close relations in the sagittal view (17). The spinopelvic relations at various body positions have been increasingly found relevant in surgical planning. Take THA as an example, where acetabular cup placement is essential to postoperative joint stability. An increasing number of studies have demonstrated individual spinopelvic relations in the lateral view as a preferred reference for cup orientation (6, 18). When one stands up or sits down, his or her rotated pelvis drives the anterior pelvic plane to shift, and the cup becomes inconsistent with the proposed safe zone (3, 19). This explains why some patients after THA with concomitant spinal diseases experience prosthetic impingement or dislocation or premature prosthetic failure due to edge loading and accelerated wearing (20, 21). In addition, when the surgery does not factor in the individual spinopelvic relations, the risk of hip spine syndrome increases because of compensatory lumbar motions (22).

The past decade has witnessed great research efforts. Providing a systematic and in-depth review on sagittal spinopelvic balance, Lazennec et al. (1) stated that spinal surgeons and THA specialists should comprehensively assess patients and their unique spinal–pelvic–femoral complex. Dorr et al. (18) investigated two structural issues of spinopelvic balance, spinal stiffness and hypermobility, and developed a classification system and THA solution for each class. Nevertheless, an elevated risk of impingement was present after surgery in nine patients with malpositioned cups and seven with pathological imbalance. The authors cited ignoring clinical conditions while emphasizing radiological data as the critical limitation of the study. Tang et al. (23) developed an algorithm for an individualized safe zone for prosthetic placement with mathematical modeling developed from a small cohort. This algorithm, however, is of limited value in clinical use as the range of motion criteria of standing position was also adopted for sitting, and the dynamic motion of the spine and pelvis during position change was not delineated. Therefore, the spinopelvic dynamics has yet to be clarified, and the answer to accurate surgical planning remains elusive.

Robust in predicting nonlinear relationships, the ANN may reveal underlying correlations among research subjects (24). For example, Galloway et al. predicted hypokalemia with an analytic model based on artificial intelligence, achieving 91% sensitivity and 72% specificity (25). Likewise, Fei et al. obtained 87.5% sensitivity and 84.43% specificity in predicting acute lung injury through an ANN model built upon 217 patients with severe acute pancreatitis (26). Recently, DeepMind's Alphafold2 has been reported with remarkable accuracy, with a potential role in forecasting the structure of almost any protein that human cells express and searching for drug targets (27).

Composed of musculoskeletal and ligament structures and commanded by neuromuscular interaction, the spinopelvic system moves within the limit of anatomy and biomechanics regardless of the health status of individuals (28). It is thus logical that the spinopelvic mechanism is characterized by an essential correlation of measurements between standing and sitting positions. This concept is corroborated by the Pearson analysis of this study, where sitting measurements were found correlated with standing LL and LT. Interestingly, adjacent to the pelvis and more adaptable than the thoracic spine, the lumbar has been acknowledged with a pivotal role in the spinopelvic balance in many studies. Therefore, future research designs should lay more emphasis on lumbar lordosis.

The outcomes of the prediction model in this study were PT, the angle between the line connecting the midpoint of the sacral plate to the center of the bifemoral heads and the plumb line, and SS, the angle between the sacral plate and the horizontal. Both measures shall be acute angles and function to describe pelvic motion tied to spinal motion as PT increases and SS decreases when the pelvis tilts posteriorly. The best model concluded from this study achieved 78.48% and 77.54% accuracy for sitting PT and SS, which is robust given the small sample utilized in the ANN. Meanwhile, the PT and SS test sets observed a disparity between the projected and the actual measurements, which could be ascribed to the small sample size or inherent error in manual measurement, though senior radiologists obtained the measurements. The manual error might be overcome in future studies with results from Weng et al. (29), where computerized measurement of AI technology scored an absolute error of 1.18 mm with a speed of 0.2 s for each film for 990 patients. In addition, PI, another outcome of prediction in the study, represents the sagittal pelvic profile and has been proved constant and independent of pelvic position after skeletal maturity (30). Built upon a small cohort, our best model still reached 95.99% accuracy in predicting sitting PI, suggesting high reliability of the model.

This article presents an innovative method in predicting changes in the sagittal parameters of the spinopelvic structure in various pelvic positions, a model built upon standing lateral radiographs of the entire spine, pelvis, and lower extremities. In particular, the model yields unprecedented accuracy of how the pelvic tilt changes as the pelvic moves, providing grounds for future studies of incremental depth. This study, however, was not immune to limitations; for example, it observed only a small number of healthy volunteers, which might not reflect the conditions of elder patients undergoing THA. To tailor the model to clinical practice, the research team will modify the model in a larger pool of data with computerized measurement technology, higher modeling complexity, and diminished overfitting. The model can also be expanded to include bidirectional change of the spinopelvic structure between standing and sitting positions and the dynamics of the entire motion cycle using motion capture systems.

## Data Availability

The original contributions presented in the study are included in the article/Supplementary Material, further inquiries can be directed to the corresponding author/s.

## References

[1] Lazennec J Brusson A Rousseau M. Lumbar-pelvic-femoral balance on sitting and standing lateral radiographs. Orthop Traumatol. (2013) 99(1):S87–S103. 10.1016/j.otsr.2012.22712.003 23375267

[2] Lewinnek GE Lewis J Tarr R Compere C Zimmerman J. Dislocations after total hip-replacement arthroplasties. J Bone Joint Surg Am. (1978) 60(2):217–20. 10.2106/00004623-197860020-00014 641088

[3] Philippot R Wegrzyn J Farizon F Fessy M-H. Pelvic balance in sagittal and lewinnek reference planes in the standing, supine and sitting positions. Orthop Traumatol. (2009) 95(1):70–6. 10.1016/j.otsr.2008.01.001 19251240

[4] DelSole EM Vigdorchik JM Schwarzkopf R Errico TJ Buckland AJ. Total hip arthroplasty in the spinal deformity population: does degree of sagittal deformity affect rates of safe zone placement, instability, or revision? J Arthroplasty. (2017) 32(6):1910–7. 10.1016/j.arth.2016.12.039 28153459

[5] McKnight BM Trasolini NA Dorr LD. Spinopelvic motion and impingement in total hip arthroplasty. J Arthroplasty. (2019) 34(7):S53–6. 10.1016/j.arth.2019.01.033 30773360

[6] Rivìere C Lazic S Villet L Wiart Y Allwood SM Cobb J. Kinematic alignment technique for total hip and knee arthroplasty: the personalized implant positioning surgery. EFORT Open Rev. (2018) 3(3):98–105. 10.1302/2058-5241.3.170022 29657851 PMC5890135

[7] Liang H Tsui BY Ni H Valentim CC Baxter SL Liu G Evaluation and accurate diagnoses of pediatric diseases using artificial intelligence. Nat Med. (2019) 25(3):433–8. 10.1038/s41591-018-0335-9 30742121

[8] Thakkar A Patel D Shah P. Pearson Correlation coefficient-based performance enhancement of vanilla neural network for stock trend prediction. Neural Comput Appl. (2021) 33(24):16985–7000. 10.1007/s00521-021-06290-2

[9] Pan F Wen H Gao X Pu H Pang Z. Clone detection based on bpnn and physical layer reputation for industrial wireless cps. IEEE Trans Ind Inform. (2020) 17(5):3693–702. 10.1109/TII.2020.3028120

[10] Peng Y Nagata MH. An empirical overview of nonlinearity and overfitting in machine learning using COVID-19 data. Chaos Solitons Fractals. (2020) 139:110055. 10.1016/j.chaos.2020.110055 32834608 PMC7324351

[11] Wang J Chen P Zheng N Chen B Principe JC Wang F-Y. Associations between mse and ssim as cost functions in linear decomposition with application to bit allocation for sparse coding. Neurocomputing. (2021) 422:139–49. 10.1016/j.neucom.2020.10.018

[12] Ferrari F Sigmund O. A new generation 99 line matlab code for compliance topology optimization and its extension to 3d. Struct Multidiscipl Optim. (2020) 62(4):2211–28. 10.1007/s00158-020-02629-w

[13] Petetin H Bowdalo D Soret A Guevara M Jorba O Serradell K Meteorology-normalized impact of the COVID-19 lockdown upon no 2 pollution in Spain. Atmos Chem Phys. (2020) 20(18):11119–41. 10.5194/acp-20-11119-2020

[14] Andrews DF. A robust method for multiple linear regression. Technometrics. (1974) 16:523–31. 10.1080/00401706.1974.10489233

[15] Hans C. Elastic net regression modeling with the orthant normal prior. J Am Stat Assoc. (2011) 106:1383–93. 10.1198/jasa.2011.tm09241

[16] Chen PH Fan RE Lin CJ. A study on smo-type decomposition methods for support vector machines. IEEE Trans Neural Netw. (2006) 17:893–908. 10.1109/TNN.2006.875973 16856653

[17] Vialle R Levassor N Rillardon L Templier A Skalli W Guigui P. Radiographic analysis of the sagittal alignment and balance of the spine in asymptomatic subjects. JBJS. (2005) 87(2):260–7. 10.2106/JBJS.D.02043 15687145

[18] Stefl M Lundergan W Heckmann N McKnight B Ike H Murgai R Spinopelvic mobility and acetabular component position for total hip arthroplasty. Bone Joint J. (2017) 99(1 Suppl A):37–45. 10.1302/0301-620X.99B1.BJJ-2016-0415.R1 28042117

[19] Lazennec JY Thauront F Robbins CB Pour AE. Acetabular and femoral anteversions in standing position are outside the proposed safe zone after total hip arthroplasty. J Arthroplasty. (2017) 32(11):3550–6. 10.1016/j.arth.2017.06.023 28697862

[20] An VV Phan K Sivakumar BS Mobbs RJ Bruce WJ. Prior lumbar spinal fusion is associated with an increased risk of dislocation and revision in total hip arthroplasty: a meta-analysis. J Arthroplasty. (2018) 33(1):297–300. 10.1016/j.arth.2017.08.040 28974376

[21] Buckland A Puvanesarajah V Vigdorchik J Schwarzkopf R Jain A Klineberg EO Dislocation of a primary total hip arthroplasty is more common in patients with a lumbar spinal fusion. Bone Joint J. (2017) 99(5):585–91. 10.1302/0301-620X.99B5.BJJ-2016-0657.R1 28455466

[22] Parvizi J Pour AE Hillibrand A Goldberg G Sharkey PF Rothman RH. Back pain and total hip arthroplasty: a prospective natural history study. Clin. Orthop. Relat. Res. (2010) 468(5):1325–30. 10.1007/s11999-010-1236-5 20127429 PMC2853644

[23] Tang H Li Y Zhou Y Wang S Zhao Y Ma Z. A modeling study of a patient-specific safe zone for tha: calculation, validation, and key factors based on standing and sitting sagittal pelvic tilt. Clin. Orthop. Relat. Res. (2022) 480(1):191–205. 10.1097/CORR.0000000000001923 34495893 PMC8673979

[24] He Y Zhao M Xu T Li S Tian H Li W. Novel cross LSTM for predicting the changes of complementary pelvic angles between standing and sitting. J Biomed Inform. (2022) 128:104036. 10.1016/j.jbi.2022.104036 35219883

[25] Galloway CD Valys AV Petterson FL Gundotra VP Treiman DL Albert DE Noninvasive detection of hyperkalemia with a smartphone electrocardiogram and artificial intelligence. J Am Coll Cardiol. (2018) 71(11S):A272–A272. 10.1016/S0735-1097(18)30813-1

[26] Fei Y Gao K Li W-q. Artificial neural network algorithm model as powerful tool to predict acute lung injury following to severe acute pancreatitis. Pancreatology. (2018) 18(8):892–9. 10.1016/j.pan.2018.09.007 30268673

[27] Baek M DiMaio F Anishchenko I Dauparas J Ovchinnikov S Lee GR Accurate prediction of protein structures and interactions using a 3-track network. Science. (2021) 373(6557):871–76. doi:10.1126/science.abj8754 34282049 10.1126/science.abj8754 PMC7612213

[28] Janssen MM Drevelle X Humbert L Skalli W Castelein RM. Differences in male and female spino-pelvic alignment in asymptomatic young adults: a three-dimensional analysis using upright low-dose digital biplanar x-rays. Spine. (2009) 34(23):E826–32. 10.1097/BRS.3200b013e3181a9fd85 19927088

[29] Weng C-H Wang C-L Huang Y-J Yeh Y-C Fu C-J Yeh C-Y Artificial intelligence for automatic measurement of sagittal vertical axis using resunet framework. J Clin Med. (2019) 8(11):1826. 10.3390/jcm8111826 PMC6912675 31683913

[30] Diebo BG Lafage V Schwab F. Pelvic incidence: the great biomechanical effort. Spine. (2016) 41:S21–2. 10.1097/brs.0000000000001430 27015063

